# Investigation of mortality rate and management of injuries caused by driving accidents in Imam Khomeini hospital of Songhor during years in 2001-2017

**Published:** 2019-08

**Authors:** Hamid Ojaghi, Reza Bayat, Vida Saniee

**Affiliations:** ^*a*^Local Health Center of Songhor, Kermanshah University of Medical Sciences, Kermanshah, Iran.

## Abstract

**Background:**

The purpose of this study was to determine the mortality rate and management of injuries caused by driving accidents in Imam Khomeini Hospital in Songhor during 2001-2017.

**Methods:**

This is a cross-sectional descriptive-analytic study. In this study, all of the randomly assigned patients who referred to Imam Khomeini Hospital in Songhor during the years 2001-2017 were enrolled in the census method and consisted of all the subjects of the study. Data were collected using a form designed by the Prevention and Combating of Health Centers. Data were analyzed using SPSS version 20 software.

**Results:**

The mean age of the dead people was 35.5. The motorcycles share in accidents were 72% in 2001 which were decreased to 41.5% at 2017. Most motorcycle accidents are related to the warmer months of the year.

**Conclusions:**

Motorcycles are more commonly used than other vehicles in Songhor, especially in the agricultural season and the warmer months of the year. The people at age group of 15 to 35 years, has the most share in traffic accidents, whose educational needs should be addressed in coordination with relevant organizations. In order to reduce accident injuries in the at-risk age group, the city's motorcycling track should be activated.

**Keywords:**

Imam Khomeini hospital, Safe Community, Road injury prevention

